# Green spaces, subjective health and depressed affect in middle-aged and older adults: a cross-country comparison of four European cohorts

**DOI:** 10.1136/jech-2020-214257

**Published:** 2021-01-26

**Authors:** J Mark Noordzij, Marielle A Beenackers, Joost Oude Groeniger, Erik Timmermans, Basile Chaix, Dany Doiron, Martijn Huisman, Irina Motoc, Milagros Ruiz, Rita Wissa, Mauricio Avendano, Frank J van Lenthe

**Affiliations:** 1 Public Health, Erasmus Medical Center, Rotterdam, The Netherlands; 2 Department of Epidemiology and Biostatistics, Amsterdam UMC - Locatie VUMC, Amsterdam, The Netherlands; 3 INSERM, Institut Pierre Louis d’Épidémiologie et de Santé Publique, Sorbonne Universités, Paris, France; 4 Maelstrom Research, Research Institute of the McGill University Health Centre, Montreal, Quebec, Canada; 5 Department of Epidemiology and Biostatistics, Amsterdam UMC - Locatie VUMC, Amsterdam, Netherlands; 6 Department of Sociology, Vrije Universiteit Amsterdam, Amsterdam, The Netherlands; 7 Department of Epidemiology and Biostatistics, Amsterdam UMC - VUMC location, Amsterdam, The Netherlands; 8 Research Department of Epidemiology and Public Health, University College London, London, UK; 9 Maelstrom Research, McGill University Faculty of Medicine, Montreal, Quebec, Canada; 10 Department of Global Health and Social Medicine, King's College London School of Social Science and Public Policy, London, UK; 11 Department of Social and Behavioral Sciences, Harvard T.H. Chan School of Public Health, Harvard University, Cambridge, Massachusetts, USA; 12 Faculty of Geosciences, Utrecht University, Utrecht, The Netherlands

**Keywords:** ageing, self-rated health, mental health, depression, geography

## Abstract

**Background:**

Studies on associations between urban green space and mental health have yielded mixed results. This study examines associations of green space exposures with subjective health and depressed affect of middle-aged and older adults in four European cohorts.

**Methods:**

Data came from four Western-European and Central-European ageing cohorts harmonised as part of the Mindmap project, comprising 16 189 adults with an average age of 50–71 years. Green space exposure was based on the distance to the nearest green space and the amount of green space within 800 m buffers around residential addresses. Cohort-specific and one-step individual participant data (IPD) meta-analyses were used to examine associations of green space exposures with subjective health and depressed affect.

**Results:**

The amount of green spaces within 800 m buffers was lowest for Residential Environment and CORonary heart Disease (Paris, 15.0 hectares) and highest for Health, Alcohol and Psychosocial factors In Eastern Europe (Czech Republic, 35.9 hectares). IPD analyses indicated no evidence of an association between the distance to the nearest green space and depressed affect (OR 0.98, 95% CI 0.96 to 1.00) or good self-rated health (OR 1.01, 95% CI 0.99 to 1.02). Likewise, the amount of green space within 800 m buffers did not predict depressed affect (OR 0.98, 95% CI 0.96 to 1.00) or good self-rated health (OR 1.01, 95% CI 0.99 to 1.02). Findings were consistent across all cohorts.

**Conclusions:**

Data from four European ageing cohorts provide no support for the hypothesis that green space exposure is associated with subjective health or depressed affect. While longitudinal evidence is required, these findings suggest that green space may be less important for older urban residents.

## Introduction

Within the context of an increasingly urbanising world, contact with natural environments may play an important role in improving subjective health and mental well-being. A recent review by WHO concluded that that there are many public health benefits of urban green spaces for the general population.^1^ Evidence suggests that urban green spaces may be linked to less chronic stress and favourable lifestyle factors, such as increased levels of physical activity,^1 2^ which strongly predict physical and mental health. Other studies have shown that individuals living in urban areas with more green space have a reduced level of stress and improved well‐being compared with those with poorer availability of green space.^3 4^ Furthermore, psychoevolutionary theories suggest that mental health can be influenced through restorative functions of natural environments.^5 6^ Yet, empirical studies on the association between green spaces and health have yielded mixed findings. While some cross-sectional^7–9^ and some longitudinal studies^4 10^ have reported associations, other studies have failed to reproduce these results or reported associations opposite to those expected.^11–13^ Most of these studies tend to rely on data from only one city or several cities within one country, limiting variation in exposure. In addition, very few studies have examined whether the hypothesised benefits of green space exposure also apply to middle-aged and older adults. Some empirical studies have shown that emotional well-being might improve with age as symptoms of depression decline.^14^ As a result, ageing may be associated with greater emotional stability. In this context, the positive associations between green spaces and mental well-being may be different for middle-aged and older adults compared with younger adults. Furthermore, it has been theorised that older adults may be particularly susceptible to characteristics of the residential environment as they are likely to spend more time closer to home than younger adults.^15^

Only a handful of studies has examined the association between green space and health outcomes across different regions or countries. A recent study concluded that associations between green space exposures and mortality differed between macroEuropean regions and that the effects were more pronounced in Western-European cities.^16^ However, this study used aggregated exposure data at the city level, implying substantial measurement error. This leads to a second problem commonly associated with studies linking green space exposures to health outcomes: a lack of consistency in defining exposure measures. Markevych *et al* 17 identified this lack of consistency noting that in epidemiological studies, green space exposure generally implies the presence of some form of green space near the home, but a standardised definition for even this; simple; exposure proxy does not exist. Green space exposure is commonly defined at the neighbourhood level. These neighbourhoods can consist of census tracts or postal code areas, or more detailed individual-level exposures, such as ‘crow-fly’ or network buffers around the residential address.^18^ Census tract data are generally easy to obtain for multiple cities, and are therefore commonly used in studies that compare multiple cities within one country.^19 20^ However, census areas are often the result from arbitrarily defined boundaries used to aggregate continuous spatial features.^18 21 22^ More sophisticated individual-level buffers that offer improvements by considering the individual’s actual location are often limited to single cities or several cities within one country, but potentially offer useful benefits for international comparisons.

This study uses individual-level green space exposure data linked to harmonised outcomes from four cohorts in ten cities across three European countries to examine the association of green space with subjective health and depressed affect in older age. By applying common exposure data and individual buffers, we reduce measurement error and maximise variation in green space exposure across multiple cohorts. We fist analyse data for each cohort separately and then pool data for all cohorts using one-step individual participant data (IPD) meta-analysis. To our knowledge, this is the first study to use harmonised data from ageing cohorts across different cities and countries, linked to detailed individual-level data on green exposure using identical buffers.

## Methods

### Data

Data were obtained from four cohort studies in the Mindmap project, which brings together longitudinal studies from multiple European countries, Canada and Russia and offers an integrated database structure for analysing harmonised data from these cohorts.^23^ Data from four ageing cohorts in the Mindmap Harmonised Dataset V.2.01 release were used: Longitudinal Ageing Study Amsterdam (LASA), Health and Living Conditions of the Population of Eindhoven and Surroundings (GLOBE), Residential Environment and CORonary heart Disease (RECORD) and Health, Alcohol and Psychosocial factors In Eastern Europe (HAPIEE). These cohorts were chosen because of the availability of harmonised exposure and outcome measures. LASA is a longitudinal population-based study of the predictors and consequences of ageing in the Netherlands.^24^ The 2005 LASA I and LASA II samples of participants residing in the cities of Amsterdam, Zwolle and surrounding areas were selected for the analyses. The GLOBE study is a prospective cohort study on the role of living conditions for health in the Dutch city of Eindhoven and surrounding areas. The 2004 sample of GLOBE participants was selected for the analyses.^25^ The RECORD study was established in 2007 to investigate environmental determinants of territorial disparities in health in the Paris metropolitan areas.^26^ Data from 2007 were used for these analyses. The HAPIEE study is a cohort study that assesses the effects of dietary factors, alcohol consumption and psychosocial factors on the health of men and women aged 45–69 years in four countries of Central and Eastern Europe.^27^ The 2006 sample of HAPIEE participants from the Czech Republic was used for the analyses. More details on the selection of respondents can be found in online supplemental file 3.

10.1136/jech-2020-214257.supp1 Supplementary data

### Exposure to green space

**Figure 1:**
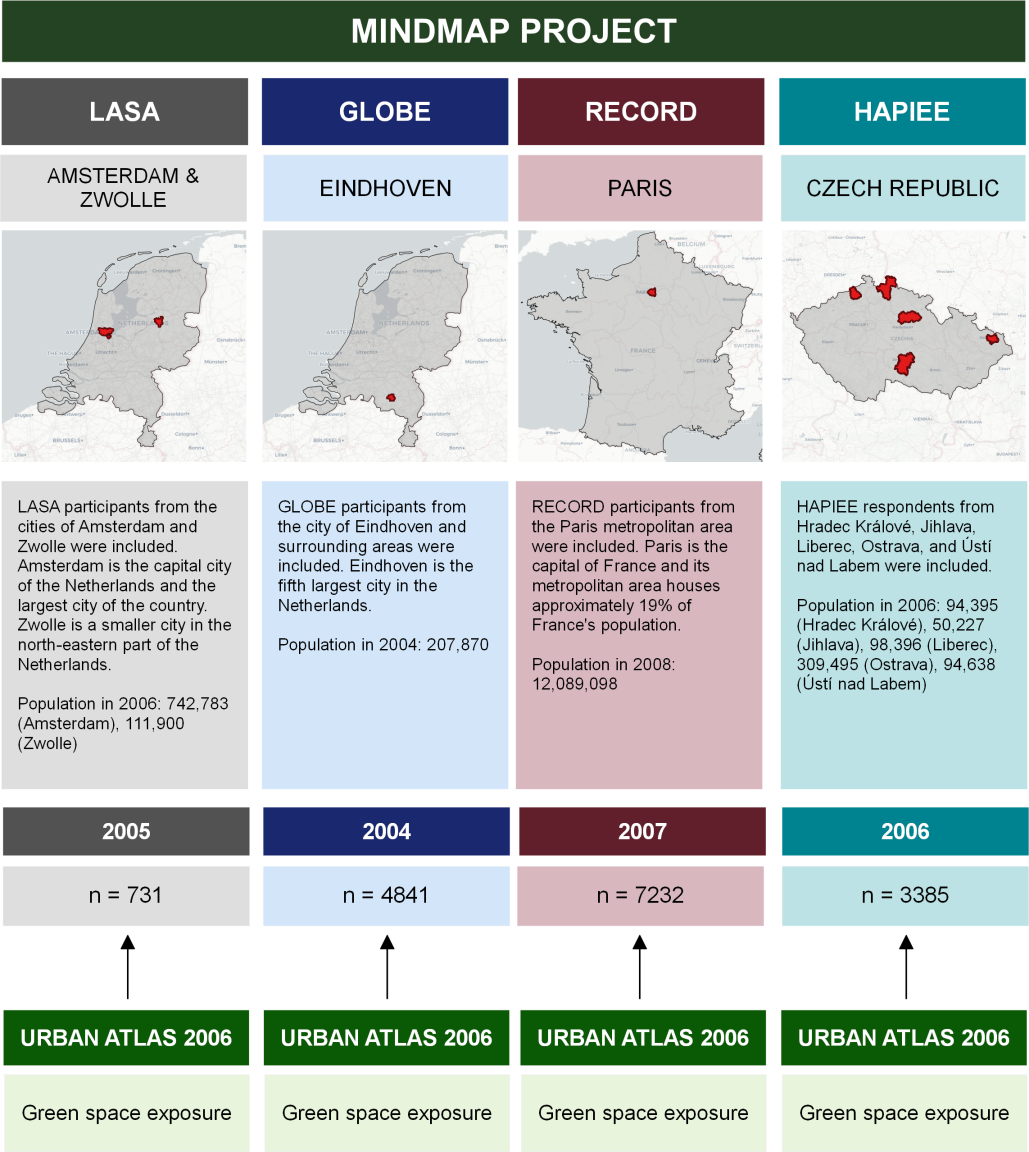
Overview of the Mindmap project and the cohorts involved in this study. Basemap: Open street map contributors & CARTO. Countries: Natural Earth Data. GLOBE, Health and Living Conditions of the Population of Eindhoven and Surroundings; HAPIEE, Health, Alcohol and Psychosocial factors In Eastern Europe; LASA, Longitudinal Ageing Study Amsterdam; RECORD, Residential Environment and CORonary heart Disease.

Geocoded respondent addresses were linked to environmental exposures as part of the Mindmap database infrastructure.^23^ Environmental exposure data were obtained from the Urban Atlas (UA) dataset. The UA is supported by the European Environment Agency and provides pan-European comparable land use and land cover data for urban areas.^28^ Land classification data were used to determine categories of green space relevant for subjective health and well-being. A category comprising all relevant green spaces (total green spaces) was used as the main exposure category. This category consisted of publicly accessible green urban areas and forest areas. More details on the green space categorisation can be found in online supplemental file 1. The straight-line distance from the participant’s residential address to the nearest point on the boundary of a green space was measured for each participant (in metres) using geographical software package QGIS.^29^ These distances were transformed to a 100 m scale to improve interpretation. Data on the amount of green space (in hectares) were calculated using Euclidian buffers of 800 m with sensitivity analyses performed on 400 m and 1000 m buffers. The amount of green space in buffers was transformed to a 10 hectares scale to improve interpretation. Cohort data for each cohort were linked to environmental exposure data from the nearest available UA wave (figure 1).

### Subjective health and depressed affect

Two measures of subjective health and well-being were available for all four cohorts within the Mindmap data release V.2.01. These included a self-reported indicator on depressed affect based on whether a participant felt sad, downhearted or blue (hereafter named ‘depressed affect’), and a dichotomous indicator of self-rated health of the participant, indicating good versus less than good health. Additionally, two subjective health and well-being outcomes that were only available for at least two cohorts were used for sensitivity analyses. These included an indicator of whether the participant had elevated psychological distress symptoms in accordance with the scale-specific threshold of psychological distress score, and an indicator of whether the participant had elevated depressive symptoms in accordance with the scale-specific threshold of depressive symptom score. More information on the harmonisation of the outcome variables can be found in online supplemental file 1.

### Statistical analysis

Modified Poisson regression models^30^ were used to estimate whether green space was associated with subjective health and depressed affect. These models included the relevant exposure and outcome measures as well as harmonised individual indicators: age, gender, employment status, retirement status, partner status (currently living with a partner) and postsecondary education as measured using the International Standard Classification of Education (ISCED). Second, we applied a one-step IPD meta-analysis. IPD meta-analyses aim to collect, check, and reanalyse individual-level data from multiple studies addressing a particular research question and can therefore be considered the gold-standard approach to evidence synthesis.^31^ We used a one-stage method that models the individual data from all studies simultaneously by pooling the data and using a hierarchical model that accounts for the clustering of participants within cohorts.^32 33^ All analyses were performed using R-studio and the Mindmap data infrastructure.^23 34^

## Results

All study cohorts consist of middle-aged and older adults with the mean age ranging from 50 (RECORD) to 71 years (LASA) (table 1). On average, the distance to the nearest green space ranged from 142 m (HAPIEE) to 267 m (RECORD). The amount of green spaces within 800 m buffers was lowest for RECORD (15.0 hectares) and highest for HAPIEE (35.9 hectares). More details on the green space exposures can be found in online supplemental file 2. Depressed affect ranged from 3.4% (LASA) to 15.2% (RECORD), and while the prevalence of good self-rated health ranged from 55.1% (HAPIEE) to 82.4% (GLOBE).

**Table 1:** Description of the study sample for each cohort

Exposures	LASA n=731	GLOBE n=4841	RECORD n=7232	HAPIEE n=3385
	Mean (SD)	Mean (SD)	Mean (SD)	Mean (SD)
Distance to nearest green space, metres	255 (268)	192 (155)	267 (220)	142 (139)
Amount of green space within 800 m buffers, hectares	17.4 (16.1)	21.9 (16.9)	15.0 (18.3)	35.9 (22.7)
Outcomes				
Depressed affect	3.4%	6.7%	15.2%	13.1%
Good self-rated health	63.9%	82.4%	52.9%	55.1%
Individual characteristics				
Age, mean (SD)	71 (9)	55 (15)	50 (12)	62 (7)
Male, %	47	48	66	46
Highest level of education completed, %				
Upper secondary or less (ISCED 0–3)	80	71	50	85
Postsecondary non-tertiary education or more (ISCED 4–8)	20	29	50	15
Employment status, %				
Currently not in paid employment	82	55	39	52
Currently in paid employment	18	45	61	48
Retirement status, %				
Currently not in retirement	n.a.	69	82	35
Currently in retirement	n.a.	31	18	65
Partner status, %				
Currently not married or living with partner	36	21	36	24
Currently married or living with partner	64	79	64	76

GLOBE, Health and Living Conditions of the Population of Eindhoven and Surroundings; HAPIE, Health, Alcohol and Psychosocial factors In Eastern Europe; LASA, Longitudinal AgingAgeing Study Amsterdam; RECORD, Residential Environment and CORonary heart Disease.

Table 2 summarises results from the modified Poisson models for each cohort separately. The distance to the nearest green space was not associated with any of the outcomes in any of the cohorts. Sensitivity analyses were performed for probable caseness of depression and psychological distress, which were not available for all cohorts; estimates yielded very similar results (online supplemental file 2).

**Table 2:** Modified Poisson regression models regressing subjective health and depressed affect on the distance to the nearest green space

Adjusted model*	LASA n=731	GLOBE n=4841	RECORD n=7232	HAPIEE n=3385
	Relative Risk (95% CI)	Relative Risk (95% CI)	Relative Risk (95% CI)	Relative Risk (95% CI)
Distance to nearest green space (per 100 m)				
Depressed affect	0.99 (0.81 to 1.12)	1.01 (0.94 to 1.08)	0.97 (0.95 to 1.00)	0.98 (0.91 to 1.04)
Good self-rated health	1.01 (0.97 to 1.04)	1.00 (0.98 to 1.02)	1.01 (0.99 to 1.03)	0.99 (0.96 to 1.02)

*Adjusted for age, gender, employment, retirement status, postsecondary education and partner status.

GLOBE, Health and Living Conditions of the Population of Eindhoven and Surroundings; HAPIEE, Health, Alcohol and Psychosocial factors In Eastern Europe; LASA, Longitudinal AgingAgeing Study Amsterdam; RECORD, Residential Environment and CORonary heart Disease.

The amount of green space in 800 m buffers was not associated with subjective health or depressed affect (table 3). Sensitivity analyses performed on both smaller and larger buffer sizes as well as other outcomes showed similar associations (online supplemental file 2). One-step IPD analyses that combined all cohorts yielded no evidence of associations of green space exposures with subjective health and depressed affect (table 4).

**Table 3:** Modified Poisson regression models regressing subjective health and depressed affect on the amount of green space within 800 m buffers around the residential address

Adjusted model*	LASA n=731	GLOBE n=4841	RECORD n=7232	HAPIEE n=3385
	Relative Risk (95% CI)	Relative Risk (95% CI)	Relative Risk (95% CI)	Relative Risk (95% CI)
Amount of green space within 800 m buffers (per 10 hectares)				
Depressed affect	1.01 (0.77 to 1.27)	0.98 (0.91 to 1.05)	1.01 (0.98 to 1.04)	1.01 (0.97 to 1.05)
Good self-rated health	1.00 (0.95 to 1.06)	1.01 (0.99 to 1.03)	1.00 (0.98 to 1.02)	1.01 (0.99 to 1.03)

*Adjusted for age, gender, employment, retirement status, postsecondary education and partner status.

GLOBE, Health and Living Conditions of the Population of Eindhoven and Surroundings; HAPIEE, Health, Alcohol and Psychosocial factors In Eastern Europe; LASA, Longitudinal AgingAgeing Study Amsterdam; RECORD, Residential Environment and CORonary heart Disease.

**Table 4:** One-step IPD analyses regressing subjective health and depressed affect on the distance to the nearest green space and on the amount of green space within 800 m buffers, adjusted for cohort

Adjusted model*	Pooled dataset
	Relative Risk (95% CI)
Distance to nearest green space (per 100 m)	
Depressed affect	0.98 (0.96 to 1.00)
Good self-rated health	1.01 (0.99 to 1.02)
Amount of green space within 800 m buffers (per 10 hectares)	
Depressed affect	1.00 (0.98 to 1.03)
Good self-rated health	1.00 (0.99 to 1.01)

*Adjusted for age, gender, employment, retirement status, postsecondary education, partner status and study.

IPD, individual participant data.

## Discussion

In the present study, we found no evidence of cross-sectional associations of green space exposures with subjective health, depressed affect and other measures of depressive symptoms. This finding appeared quite consistent across four cohorts with diverse settings and levels of exposure to green space. Studies conducted on the effect of green spaces on health outcomes tend to rely on data from only one city or one country, limiting variation as well as generalisability. Our study addressed this issue by including data from ten cities across four cohorts from three countries. The results for the Dutch cohorts are in line with other studies conducted in the Netherlands. For example, a study conducted in Maastricht, The Netherlands did not find associations between green spaces and self-rated health.^35^ A previous study using data from eight Dutch cohorts—including the LASA cohort—found some inconsistent associations between green space and a prevalence of depression.^19^ A previous study using the GLOBE data used very similar green space exposures, and found inconsistent associations between distance to the nearest green space and a more detailed measure of mental health.^36^

Inconsistent findings are not new in the literature on urban green spaces and health and extend beyond the Dutch context.^10 37^ While a number of reviews and meta-analyses conclude that urban green spaces can be beneficial for subjective health and well-being, other studies find no associations or even report associations opposite to those expected.^11–13^ This could be the result of variation in methodological approaches and the measurement of green spaces. In nearly all epidemiological studies, green space exposures are defined as the presence of some form of green space in the residential environment, but some studies make use of census data or postal code areas to define green space exposure, while others make use of buffers; either circular ‘crow fly’ or network-based ones. Multiple studies have shown these differences in defining green space exposures can result in variation in associations.^21 37^ Variation in geographical units and scales used to define the exposures could mask consistencies that may actually exist between different studies.

There are multiple possible interpretations for the findings of this study. First, the lack of associations in the present study may suggest that urban green space exposures have a limited influence on individuals’ subjective perceptions of their own health and mental well-being. Prior studies have focused on the impact of green spaces on outcomes such as physical activity, which may be critical for physical health outcomes, but their influence on mental health in older age may be less marked. Second, findings may also indicate that other, non-measured aspects of green spaces, such as their quality and design, might still be associated with health outcomes and be more important than the presence of green space in the residential environment. For example, a Dutch study showed that specific characteristics of green spaces, such as their size and quality, may influence the effect of green spaces on multiple outcomes.^38^ Likewise, evidence from the UK also suggests that variations in ‘ecological quality’, that is, habitat diversity and ecological functions, may determine whether green spaces have psychological restorative benefits to residents.^39^ Third, the impact of green spaces on subjective and mental health may be contingent on other, possibly intertwining, factors not measured in our study. For example, green spaces may only bring benefits if they influence risk factors associated with subjective and mental well-being, such as social interactions or exposure to harmful environmental stressors. For example, Pietilä *et al* 40 found that exposure to green spaces was associated with self-rated health, but the mechanisms that explain this association were different for suburbs compared with more urban residential areas.^40^

Aside from methodological limitations, these findings raise the possibility that green space might not be associated with the health of middle-aged and older adults. Some earlier studies have also failed to find consistent evidence that a change in green space exposure in a relatively green city improves health.^35 41^ A possible explanation for these findings might be found in what is labelled the paradox of ageing: empirical studies show that emotional well-being trends to improve with older age, while symptoms of depression decline as individuals get older.^14^ While the explanation of this age pattern is not fully understood, life span development theories, such as the socioemotional selectivity theory, suggest that older people may attach greater importance to finding emotional meaning and less importance to other goals.^42^ As a result, ageing may be associated with more positive emotions and greater emotional stability. In this context, green spaces may become less important for older people as they become less goal oriented and more focused on the regulation of emotional states. More research that explores this hypothesis is warranted.

### Strengths and limitations

The current study aims to add to the literature on the health benefits of urban green spaces by using a cross-country perspective to investigate if green spaces in the residential environment are related to subjective health and depressed affect in Western and Central European cities. Some limitations of our study should be considered. We were not able to control for other urban-environmental factors, such as residential density or neighbourhood socioeconomic status, because either these data were not available for all cities or we were not able to harmonise the data on the same spatial scale as our exposure data (ie, 800 m buffers). One of the strengths of this study is that all data are harmonised across the cohorts, enabling a valid cross-country comparison. Introducing other environmental data on different geographical scales would not only weaken this comparison, but would also introduce biases associated with the spatial configuration of neighbourhoods, the overlap of varying spatial extends and other issues of spatial misclassification.^19^

The green space data used in this study were limited to publicly accessible green spaces, such as parks and forests. These areas represent green spaces that policy makers can influence as opposed to private green spaces. However, it should be noted that the exclusion of private green spaces can potentially bias our results as they may provide functions similar to public green spaces (eg, views of nature). The green space measures in this study are based on individual-level buffers and distances around the residential address. While we consider this a strength of our study as not many studies that use data from different cities in multiple countries use such specific measures, it has to be noted that our study uses straight-line distances and so-called ‘Crow-fly’ buffers. These measures do not take accessibility of green spaces into account as there may be a physical barrier preventing access. We could not investigate whether differences exist between respondents that had recently moved to the address compared with those that already resided at the address for a longer period of time. The data collection waves of the included studies had a maximum of 2 years mismatch with the green space data. An important assumption therefore is that the green space measures used, remained relatively constant over 2 years. A violation of this assumption may slightly bias our findings in an unpredictable direction. However, a previous study using similar exposure data and health outcomes from the GLOBE cohort, found that the majority of respondents had no, or very small changes, in green space exposure over a period of 10 years.^36^ Finally, as this is a cross-sectional study, we do not know whether the participants’ health status preceded or proceeded the exposure to green space.

We were able to control for a number of relevant individual characteristics, such as employment and education, but not all of these characteristics were available for all cohorts. For example, data on household income had to be excluded from the analyses as it was not available for the HAPIEE cohorts and contained a relatively large number of missing values in the GLOBE cohort. We conducted additional analyses with only the LASA and RECORD cohorts that included data on household income, but these yielded very similar results to those presented here. For the HAPIEE cohort we had to resort to using the post-secondary education from the baseline data wave (2002) as this variable was not available for the wave that was used in the analyses (2006). However, it is unlikely that this has influenced the results as education status rarely changes in middle-aged and older adults.

The Mindmap project makes use of retrospective harmonisation of cohort data, which means that study variables are harmonised after they have been collected. While this is a great way to make comparisons between cohorts possible, it does inherently come with the limitation that some detail is lost in the harmonisation. For example, the LASA wave used in this study only contained data on early retirement, while the other cohorts included data on general retirement status. Such harmonisation choices lead to an inevitable loss in sensitivity in covariates as well as in the outcomes. Furthermore, while the harmonisation makes a comparison of the associations possible, prevalences might not be comparable. This, however, is unlikely to be a major issue when comparing associations between variables across cohorts. More prospective harmonisation would alleviate these limitations and therefore make more comparisons between cohorts possible.

## Conclusion

The present study did not find evidence of associations of green space exposures with subjective health and depressed affect in middle-aged and older adults. A possible interpretation is that distance to or amount of green space near the home may not be the most important feature for subjective health and mental well-being, but that other factors, such as the quality of green space, may be more important. However, results also suggest that green spaces may be only weak predictors of subjective health and mental well-being in older people, who may benefit less from the proximity to green spaces than other age groups. More research using longitudinal data and examining confounding is needed to better understand how green spaces and subjective health and mental well-being relate.

**What is already known on this subject**

- Urban green spaces are often linked to better health through pathways such as restoration of stress and attentional fatigue, and improved physical activity. However, empirical studies of single cities or countries have revealed mixed and inconsistent findings and do not focus specifically on older urban residents.

**What this study adds**

- Our study combines data from four ageing cohorts from West and Central Europe enriched with harmonised green space exposure data. Findings provide no evidence of an association between green space exposure and subjective health and depressed affect across different urban contexts with extensive control for confounding.

## Data Availability

Data may be obtained from a third party and are not publicly available. The datasets generated for the Mindmap project are not publicly available due to study participant privacy considerations. However, data access can be requested from the individual cohort studies via the respective data access procedures in place.
