# Supplementary material for: Green spaces, subjective health and depressed affect in middle-aged and older adults: a cross-country comparison of four European cohorts
Source: J Epidemiol Community Health. 2021 Jan 26;75(5):470–6. doi: 10.1136/jech-2020-214257 (PMC8053337; doi:10.1136/jech-2020-214257)
Supplement: Supplementary data [file jech-2020-214257supp001.pdf]

SUPPLEMENTARY FILE 1: More details on the harmonization of the outcome variables and the green space exposure measures

MAIN OUTCOMES				
	**LASA**	**GLOBE**	**RECORD**	**HAPIEE**
**Self-rated health**	Dichotomous indicator of self-assessed health of the participant, indicating good versus less than good health. Based on an original categorization of very good, good, moderate, sometimes good, sometimes bad, and bad self-rated health.	Dichotomous indicator of self-assessed health of the participant, indicating good versus less than good health. Based on an original categorization of excellent, very good, good, fair and poor self-rated health.	Dichotomous indicator of self-assessed health of the participant, indicating good versus less than good health. Based on a score between 0 (terrible) and 10 (excellent) self-rated health, with all scores >7 labeled as good.	Dichotomous indicator of self-assessed health of the participant, indicating good versus less than good health. Based on an original categorization of very good, good, average, poor and very poor self-rated health.
**Feels sad, downhearted, or blue**	Based on a 4-point ('rarely or none of the time', 'some or a little of the time', 'occasionally or a moderate amount of time', 'most or all of the time') item response to "I felt sad" from the CES-D scale, 20-item version. Participants who responded 'occasionally or a moderate amount of time' or 'most or all of the time' were defined as feeling sad, downhearted or blue.	Based on a 6-point ('all of the time', 'most of the time', 'a good bit of the time', 'some of the time', 'a little of the time', 'none of the time') item response to "How much of the time during the past 4 weeks, have you felt downhearted and blue?" from the MHI-5 scale. Participants who responded 'all of the time' to 'a good bit of the time' were defined as feeling sad, downhearted or blue.	Based on a yes/no item response to "I feel sad at present" from the QD2A depression scale.	Based on a 4-point ('less than 1 day', '1-2 days', '3-4 days', '5-7 days') item response to "I felt sad" from the CES-D scale, 20-item version. Participants who responded '3-4 days' or '5-7 days' were defined as feeling sad, downhearted or blue.
**ADDITIONAL OUTCOMES***				
	**LASA**	**GLOBE**	**RECORD**	**HAPIEE**
**Probable caseness of depression**	Participants with CESD-20 scores of 16 or higher (out of a possible 60) were defined as cases as per the scale-specific threshold [1].	n.a.	Participants with QD2A depression scores of 7 or higher (out of a possible 13) were defined as cases as per the scale-specific threshold [2].	Participants with CESD-20 scores of 16 or higher (out of a possible 60) were defined as cases as per the scale-specific threshold [1].
**Probable caseness of psychological distress**	The MHI-2 scores (2-12) in LASA were transformed to a scale ranging from 0 to 100. As there is no established cut-off score for the MHI-2, participants whose scores ranged from 50 to 100 were classified as cases.	The MHI-4 scores (4-24) in GLOBE were transformed to a scale ranging from 0 to 100. As there is no established cut-off score for the MHI-4, participants whose scores ranged from 50 to 100 were classified as cases.	n.a.	n.a.

* Additional outcomes were only used for sensitivity analyses.

MORE DETAILS ON THE GREEN SPACE EXPOSURE MEASURES

The Urban Atlas service offers a high-resolution land use map of urban areas in the European Union. It contains data derived from Earth Observation (EO) data backed by other reference data, such as Commercial Off-The-Shelf (COTS) or Open Street Map (OSM) navigation data and topographic maps [7]. Polygons of specific land use classes need to have a minimal size to be included in a certain category. This size is known as the Minimal Mapping Unit (MinMU). Included in the total green space category are:

GREEN URBAN AREAS

MinMU 0.25 ha, Minimum width: 10 m

- Public green areas for predominantly recreational use such as gardens, zoos, parks, castle parks.
- Suburban natural areas that have become and are managed as urban parks.
- Forests or green areas extending from the surroundings into urban areas are mapped as green urban areas when at least two sides are bordered by urban areas and structures, and traces of recreational use are visible.

Not included are:

- Private gardens within housing areas;
- Cemeteries;
- Buildings within parks, such as castles or museums;
- Patches of natural vegetation or agricultural areas enclosed by built-up areas without being managed as green urban areas.

FOREST (NATURAL AND PLANTATION)

MinMU 1 ha

- With ground coverage of tree canopy > 30%, tree height > 5 m, including bushes and shrubs at the fringe of the forest;
- Included are plantations such as Populus plantations, Christmas tree plantations;
- Forest regeneration / re-colonisation: clear cuts, new forest plantations.

Not included are:

- Forests within urban areas and/or subject to high human pressure (see green urban areas).

More details on the land use classification used in the Urban Atlas can be found in the official mapping guide: https://land.copernicus.eu/user-corner/technical-library/urban-atlas-mapping-guide-2006

Distance to the nearest green space in meters										
**LASA**										
Mean (SD)	255 (268)									
Min. – max.	0 – 2100									
Distribution (percentiles)	**0-10**	**10-20**	**20-30**	**30-40**	**40-50**	**50-60**	**60-70**	**70-80**	**80-90**	**90-100**
	0-40	40-77	77-107	107-143	143-180	180-236	236-289	289-363	363-549	549-2100
**GLOBE**										
Mean (SD)	192 (159)									
Min. – max.	0 – 1528									
Distribution (percentiles)	**0-10**	**10-20**	**20-30**	**30-40**	**40-50**	**50-60**	**60-70**	**70-80**	**80-90**	**90-100**
	0-38	38-68	68-99	99-129	129-159	159-195	195-240	240-297	297-381	381-1528
**RECORD**										
Mean (SD)	267 (217)									
Min. – max.	0 – 1629									
Distribution (percentiles)	**0-10**	**10-20**	**20-30**	**30-40**	**40-50**	**50-60**	**60-70**	**70-80**	**80-90**	**90-100**
	0-50	50-93	93-136	136-178	178-220	220-264	264-322	322-397	397-539	539-1629
**HAPIEE**										
Mean (SD)	142 (139)									
Min. – max.	0 - 1167									
Distribution (percentiles)	**0-10**	**10-20**	**20-30**	**30-40**	**40-50**	**50-60**	**60-70**	**70-80**	**80-90**	**90-100**
	0-19	19-35	35-58	58-80	80-107	107-135	135-172	172-218	218-298	298-1167
Amount of green space in 800-meter buffers in hectares*										
**LASA**										
Mean (SD)	17.4 (16.1)									
Min. – max.	0 – 104.8									
Distribution (percentiles)	**0-10**	**10-20**	**20-30**	**30-40**	**40-50**	**50-60**	**60-70**	**70-80**	**80-90**	**90-100**
	0-1.2	1.2-2.9	2.9-6.1	6.1-9.1	9.1-12.0	12.0-18.4	18.4-24.1	24.1-29.6	29.6-38.1	38.1-104.8
**GLOBE**										

Mean (SD)	21.9 (16.9)									
Min. – max.	0 – 130.6									
Distribution (percentiles)	**0-10**	**10-20**	**20-30**	**30-40**	**40-50**	**50-60**	**60-70**	**70-80**	**80-90**	**90-100**
	0-5.9	5.9-9.2	9.2-11.9	11.9-14.6	14.6-17.8	17.8-21.8	21.8-25.8	25.8-31.0	31.0-41.0	41.0-130.6
**RECORD**										
Mean (SD)	15.0 (18.3)									
Min. – max.	0 – 189.3									
Distribution (percentiles)	**0-10**	**10-20**	**20-30**	**30-40**	**40-50**	**50-60**	**60-70**	**70-80**	**80-90**	**90-100**
	0-1.6	1.6-3.6	3.6-5.1	5.1-6.6	6.6-8.7	8.7-10.9	10.9-14.4	14.4-22.0	22.0-38.0	38.0-189.3
**HAPIEE**										
Mean (SD)	35.9 (22.7)									
Min. – max.	0 – 166.0									
Distribution (percentiles)	**0-10**	**10-20**	**20-30**	**30-40**	**40-50**	**50-60**	**60-70**	**70-80**	**80-90**	**90-100**
	0-11.2	11.2-16.0	16.0-20.6	20.6-27.0	27.0-32.1	32.1-38.7	38.7-44.4	44.4-51.3	51.3-65.6	65.6-166.0

* The total area size of an **800-meter** buffer is approximately **201 hectares**. A decrease or increase of 10 hectares corresponds to approximately **5%** of the total buffer size.

SUPPLEMENTARY FILE 2: Sensitivity analyses

Table 1: Descriptive statistics of the additional subjective health and well-being outcomes for each cohort

	LASA	GLOBE	RECORD	HAPIEE
	Mean (SD	Mean (SD)	Mean (SD)	Mean (SD)
Probable caseness of depression, % (n)	15.3% (112)	n.a.	7.7% (558)	11.1% (377)
Probable caseness of psychological distress, % (n)	17.8% (130)	8.7% (419)	n.a.	n.a.

Table 2: Modified Poisson regression models regressing the probable caseness of depression and psychological distress on the distance to the nearest green space

	LASA	GLOBE	RECORD	HAPIEE
**Adjusted model***	**RR (95% CI)**	**RR (95% CI)**	**RR (95% CI)**	**RR (95% CI)**
Distance to nearest green space (per 100m)				
Probable caseness of depression	1.02 (0.95 – 1.08)	n.a.	0.98 (0.94 – 1.01)	1.00 (0.93 – 1.08)
Probable caseness of psychological distress	1.04 (0.98 – 1.10)	0.97 (0.91 – 1.03)	n.a.	n.a.

*adjusted for age, gender, employment, retirement status, post-secondary education, and partner status.

Table 3: Modified Poisson regression models regressing the probable caseness of depression and psychological distress on the amount of green space within 800-meter buffers

	LASA	GLOBE	RECORD	HAPIEE
**Adjusted model***	**RR (95% CI)**	**RR (95% CI)**	**RR (95% CI)**	**RR (95% CI)**
Amount of green space within 800-meter buffers (per 10 hectares)				
Probable caseness of depression	1.02 (0.92 – 1.15)	n.a.	1.00 (0.96 – 1.05)	0.99 (0.95 – 1.04)
Probable caseness of psychological distress	1.02 (0.91 – 1.13)	0.98 (0.92 – 1.03)	n.a.	n.a.

*adjusted for age, gender, employment, retirement status, post-secondary education, and partner status.

Table 4: Modified Poisson regression models regressing subjective health and well-being outcomes on the amount of green spaces in 400 and 1000 meter buffers

	LASA	GLOBE	RECORD	HAPIEE
**Adjusted model***	**RR (95% CI)**	**RR (95% CI)**	**RR (95% CI)**	**RR (95% CI)**
Amount of green space within 400-meter buffers (per 10 hectares)				
Depressed affect	1.10 (0.42 – 2.44)	0.93 (0.73 – 1.18)	1.06 (0.92 – 1.21)	1.01 (0.87 – 1.17)
Good self-rated health	0.99 (0.80 – 1.21)	1.03 (0.97 – 1.10)	1.00 (0.94 – 1.06)	1.03 (0.96 – 1.11)
**Adjusted model***	**RR (95% CI)**	**RR (95% CI)**	**RR (95% CI)**	**RR (95% CI)**
Amount of green space within 1000-meter buffers (per 10 hectares)				
Depressed affect	0.95 (0.78 – 1.13)	0.99 (0.94 – 1.03)	1.01 (0.99 – 1.03)	1.01 (0.98 – 1.04)
Good self-rated health	1.00 (0.96 – 1.04)	1.00 (0.99 – 1.02)	1.00 (0.99 – 1.01)	1.00 (0.99 – 1.02)

*adjusted for age, gender, employment, retirement status, post-secondary education, and partner status.

SUPPLEMENTARY FILE 3: Selection of respondents

Selection LASA respondents

Response rate	"The initial response rate [calculated following guidelines from the American Association for Public Opinion Research (AAPOR3)] is 60% (n = 3805), and the cooperation rate is 62%. The response rate is defined here as the number of complete and partial interviews with persons divided by the total number of eligible persons in the sample plus a fraction of those persons who were in the sample but of whom eligibility could not be determined. The cooperation rate is defined as the proportion of completed interviews in the number of contacted eligible persons [3]."

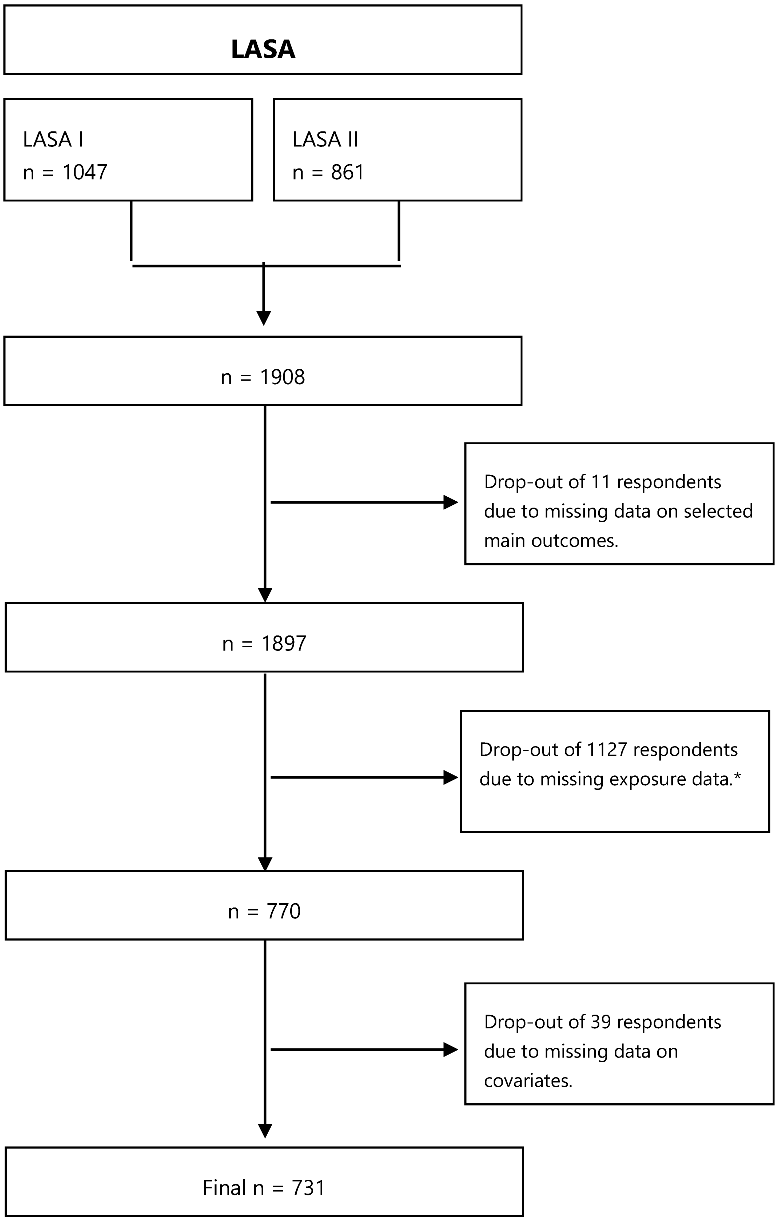

* Exposure data was not available for one of the three cities that LASA wave 2005/2006 was conducted in.

FIGURE 2: selection of GLOBE respondents

Response rate	The GLOBE 2004 data collection wave had a response rate of 64.4%. Information became available for 6377 persons [4].

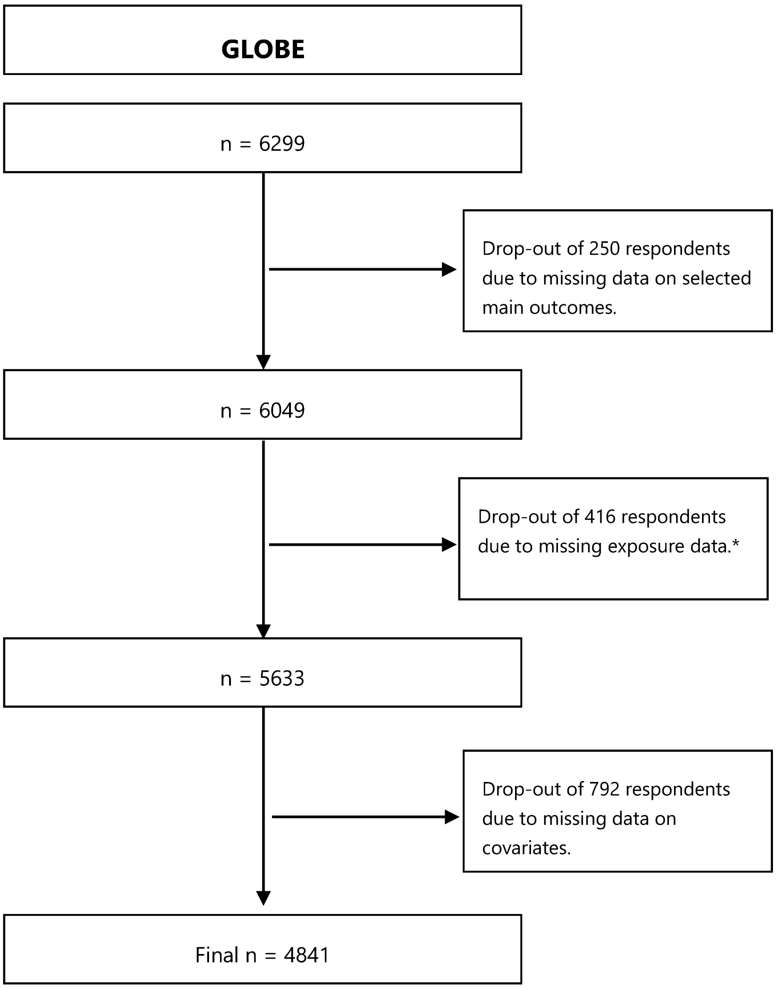

* GLOBE respondents living outside of the Eindhoven area were excluded due to a lack of available environmental data.

FIGURE 3: selection of RECORD respondents

Response rate	Of the persons selected for participation, 83.6% accepted to participate and completed the data collection protocol. Overall, 7290 participants were recruited between March 2007 and February 2008. [5].

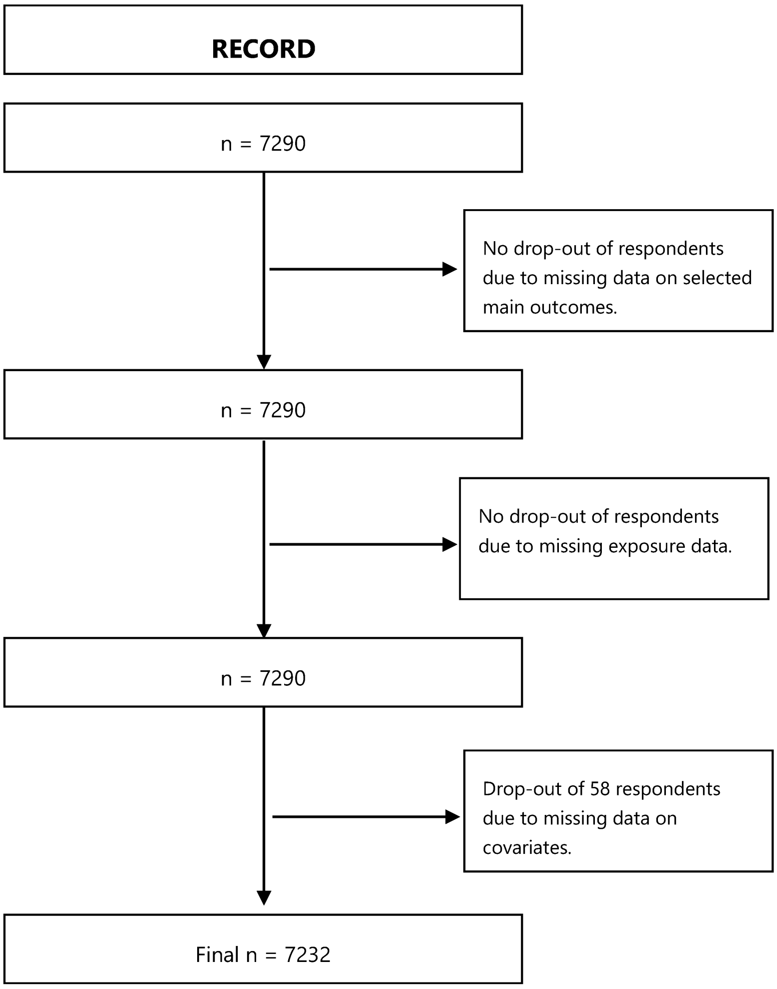

FIGURE 4: selection of HAPIEE respondents

Response rate	The HAPIEE Czech Republic cohort had a response rate of 55% for a total of 8,856 respondents [6].

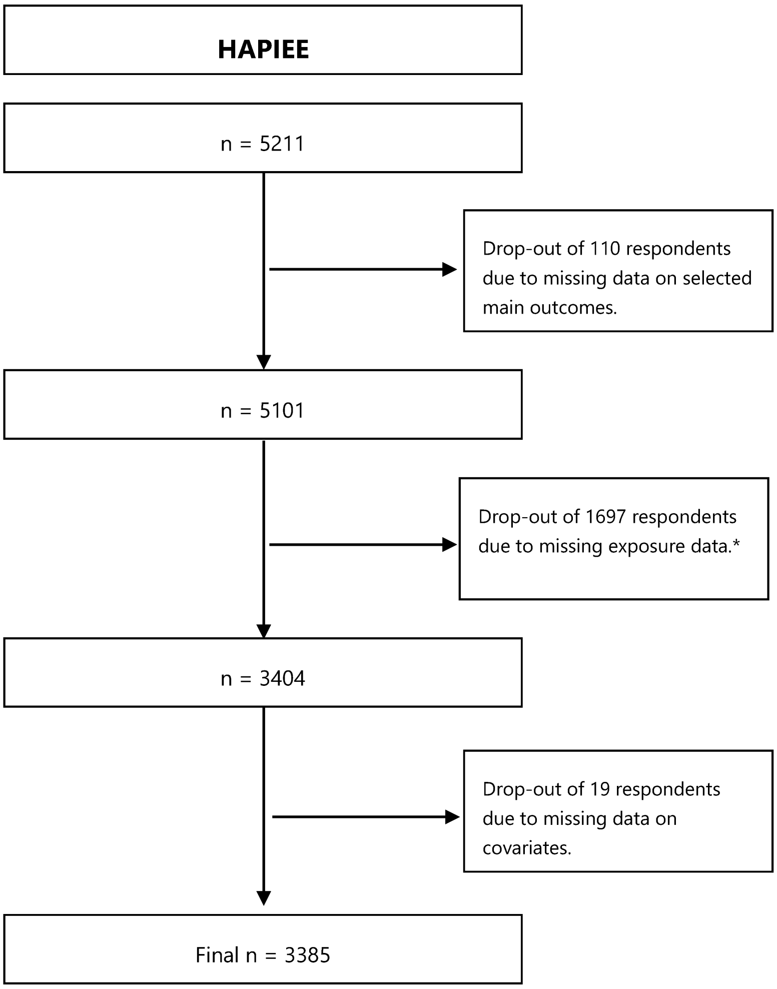

* Exposure data was not available for all cities in the HAPIEE Czech cohort.

REFERENCES

1. Weissman MM, Sholomskas D, Pottenger M, et al. Assessing depressive symptoms in five psychiatric populations: a validation study. Am J Epidemiol 1977;106(3):203-14. doi: 10.1093/oxfordjournals.aje.a112455
2. Pichot P. A Self-Report Inventory on Depressive Symptomatology (QD2) and Its Abridged Form (QD2A). In: Sartorius N, Ban TA, eds. Assessment of Depression. Berlin, Heidelberg: Springer Berlin Heidelberg 1986:108-22.
3. Huisman M, Poppelaars J, van der Horst M, Beekman AT, Brug J, van Tilburg TG, et al. Cohort Profile: The Longitudinal Aging Study Amsterdam. International Journal of Epidemiology. 2011; 40(4): 868-76.
4. Van Lenthe FJ, Kamphuis CBM, Beenackers MA, Jansen T, Looman CWN, Nusselder WJ, et al. Cohort Profile: Understanding socioeconomic inequalities in health and health behaviours: The GLOBE study. International Journal of Epidemiology. 2013; 43(3): 721-30.
5. Chaix B, Kestens Y, Bean K, Leal C, Karusisi N, Meghiref K, et al. Cohort Profile: Residential and non-residential environments, individual activity spaces and cardiovascular risk factors and diseases--The RECORD Cohort Study. International journal of epidemiology. 2012 October; 41(5): 1283-92.
6. Peasey A, Bobak M, Kubinova R, Malyutina S, Pajak A, Tamosiunas A, et al. Determinants of cardiovascular disease and other non-communicable diseases in Central and Eastern Europe: Rationale and design of the HAPIEE study. BMC Public Health. 2006; 6(1): 255.
7. Urban Atlas (2006), Mapping guide. Online: https://land.copernicus.eu/user-corner/technical-library/urban-atlas-mapping-guide-2006.
